# Identification and Validation of Genomic Regions Associated With Charcoal Rot Resistance in Tropical Maize by Genome-Wide Association and Linkage Mapping

**DOI:** 10.3389/fpls.2021.726767

**Published:** 2021-10-08

**Authors:** Zerka Rashid, Harleen Kaur, Veerendra Babu, Pradeep Kumar Singh, Sharanappa I. Harlapur, Sudha K. Nair

**Affiliations:** ^1^International Maize and Wheat Improvement Center (CIMMYT), ICRISAT Campus, Hyderabad, India; ^2^Department of Plant Breeding and Genetics, Punjab Agricultural University, Ludhiana, India; ^3^Department of Plant Pathology, University of Agricultural Sciences, Dharwad, India

**Keywords:** GWAS–genome-wide association study, linkage (QTL) mapping, haplotype analysis, charcoal rot, maize

## Abstract

Charcoal rot is a post-flowering stalk rot (PFSR) disease of maize caused by the fungal pathogen, *Macrophomina phaseolina*. It is a serious concern for smallholder maize cultivation, due to significant yield loss and plant lodging at harvest, and this disease is expected to surge with climate change effects like drought and high soil temperature. For identification and validation of genomic variants associated with charcoal rot resistance, a genome-wide association study (GWAS) was conducted on CIMMYT Asia association mapping panel comprising 396 tropical-adapted lines, especially to Asian environments. The panel was phenotyped for disease severity across two locations with high disease prevalence in India. A subset of 296,497 high-quality SNPs filtered from genotyping by sequencing was correcting for population structure and kinship matrices for single locus mixed linear model (MLM) of GWAS analysis. A total of 19 SNPs were identified to be associated with charcoal rot resistance with *P-*value ranging from 5.88 × 10^−06^ to 4.80 × 10^−05^. Haplotype regression analysis identified 21 significant haplotypes for the trait with Bonferroni corrected *P* ≤ 0.05. For validating the associated variants and identifying novel QTLs, QTL mapping was conducted using two F_2:3_ populations. Two QTLs with overlapping physical intervals, qMSR6 and qFMSR6 on chromosome 6, identified from two different mapping populations and contributed by two different resistant parents, were co-located with the SNPs and haplotypes identified at 103.51 Mb on chromosome 6. Similarly, several SNPs/haplotypes identified on chromosomes 3, 6 and 8 were also found to be physically co-located within QTL intervals detected in one of the two mapping populations. The study also noted that several SNPs/haplotypes for resistance to charcoal rot were located within physical intervals of previously reported QTLs for Gibberella stalk rot resistance, which opens up a new possibility for common disease resistance mechanisms for multiple stalk rots.

## Introduction

Maize is cultivated on more than 180 million hectares (M ha) globally, contributing ~50% [1,117 million metric tons (MMTs)] to the global grain production (Prasanna, 2018). Asian countries have shown rapid progress in maize production and productivity and are the second largest maize producers in the world with 31% share in global maize production (Zaidi et al., 2018). China produced nearly 260.95 MMT of maize by cultivating the maize area of 41.30 M ha during 2019 (FAO., 2021). The second prime maize producing country among Asian countries is India with an estimated maize area of ~9.03 M ha in 2019 with the maize production of 27.72 M Mt at a productivity of 3.07 t/ha (FAO., 2021). In Asia, a large portion of maize (~70% of total volume) is used by the feed industry (Prasanna, 2018), and the maize demand is always increasing due to the rise in population and socio-economic growth (Shiferaw et al., 2011). Apart from feed, maize is increasingly used in industries especially in food processing industry for making additives and sweeteners (Prasanna, 2018).

Despite the substantial growth rates in terms of cultivated maize area, production, and productivity in the last few years, maize in the south and southeast Asia is largely (80%) grown as a rainfed crop that is prone to the vagaries of monsoon rains, in addition to a number of biotic and abiotic stresses in this region (Zaidi et al., 2018). Abiotic stresses like drought, heat, and waterlogging are the main stresses that have a high impact on yield loss. Compounded with these, diseases have a huge impact on grain yield, as observed in most of the countries in Asia. The most common and economically important diseases in the region are soil-borne diseases like post-flowering stalk rots (PFSR) and banded leaf and sheath blight (BLSB) and foliar diseases like Turcicum leaf blight (TLB), downy mildews (DM), common rust, and polysora rust. Due to the impact of climate change effects, maize stalk rots and ear rots are reported to become more severe and widespread (Prasanna et al., 2021). Stalk rots in maize are caused by many fungi and bacteria, most of which occur commonly in the fields and behave opportunistically by infecting senescing, injured, and stressed plants (Jackson-Ziems et al., 2014). Stalk rots caused by fungi are Fusarium stalk rot (FSR), Gibberella stalk rot (GSR), late wilt, Anthracnose stalk rot (ASR), Diplodia stalk rot (DSR), and charcoal rot (CR).

*Macrophomina phaseolina* (Tassi) Goid., which causes charcoal rot of maize, is economically one of the most important pathogens that have a wide host range, affecting more than 500 species of plants. Microsclerotia of *M. phaseolina* survive in the soil, and the infected plant remains serve as a basic source of infection for the crops. They are ubiquitous under raised soil temperature and low moisture conditions, and in moisture-less soil, they can exist for more than 10 months (Khan, 2007). Charcoal rot symptoms are distinguished by the appearance of a large number of minute black sclerotia on vascular bundles and inside the rind of the stalk, resulting in grayish black stalk color. Symptoms of charcoal rot are observed after plant reproductive growth, when the fungus spreads into the lower internode of the stalk causing soft stalk, premature drying of stalk, and lodging of plants (Khokhar et al., 2014), and hence, the economic impact of the disease is high. Disease severity is exacerbated by low soil moisture, and higher soil and air temperature (Smith and Wyllie, 1999), which are serious constraints faced under smallholder farming conditions in climate-vulnerable environments. It is distributed worldwide in the tropics and subtropics, as well as in the US northern, central, and southern regions (Wyllie, 1988). It is a serious biotic concern in Asian countries like China, India, Indonesia, Pakistan, Philippines, Thailand, and Vietnam (Sharma et al., 1993). Yield loss due to charcoal rot was estimated to be 25–32.2% in India (Kumar et al., 1996) and recorded as high as 63.5% in All India Coordinated Research Program trials (Maize AICRP., 2014). These losses can be avoided by the deployment of resistant cultivars, as chemical control to soil-borne diseases has been reported as largely ineffective, and it increases the cultivation cost of resource-constrained farmers, apart from having hazardous effects on the environment.

Resistance to CR is shown to be a polygenic trait, with additive and non-additive gene action, with significant environmental interaction (Singh and Kaiser, 1991; Krishna et al., 2013, Mir et al., 2018). Incorporating resistance to diseases like charcoal rot, which are quantitatively inherited and have significant environmental interaction, in the breeding schemes to enhance genetic gains over time, necessitates the use of all modern breeding tools and strategies. Molecular technologies are used to accelerate the breeding for disease resistance by the possibility to expand the size of breeding populations, thereby increasing selection intensity, without increasing phenotyping requirements. Genotypic information can be used to select germplasm at the early stages of selection, and the capability to increase this phenotypically untested layer will allow the total number of genotypes within a breeding program to be expanded (Cooper et al., 2014). Linkage mapping can be used to identify quantitative trait loci (QTLs), which, in turn, are the tools for selection of loci of interest in breeding crosses and hence act as a proxy to the actual trait. Among the different PFSR, QTL mapping studies have been reported for resistance to stalk rots like GSR and ASR in maize. Three moderate to major QTLs have been identified, and one among them has been fine mapped for resistance to GSR caused by *Fusarium graminearum* (Yang, 2010; Zhang et al., 2012; Ma et al., 2017). A major QTL for ASR (caused by *Colletotrichum graminicola*) was cloned and found to belong to a nucleotide-binding site-leucine-rich repeat (NBS-LRR) gene class on the long arm of chromosome 4 (Jung et al., 1994; Abad et al., 2006; Broglie et al., 2011). Molecular mapping studies for charcoal rot resistance in maize have not been reported yet, which may be attributed to several factors like limited availability of disease-resistant sources, complex nature of the disease, and possible co-infection with other stalk rot pathogens under natural conditions leading to low repeatability in trials. However, QTLs for resistance to charcoal rot caused by *M. phaseolina* have been reported in crops like sorghum (Mahmoud et al., 2018), soybean (da Silva et al., 2019), and sesame (Wang et al., 2017). Keeping in view the increasing incidences of charcoal rot of maize in South Asia and gap in knowledge on the genomic regions conferring resistance to the trait, we conducted this study to discover trait markers through genome-wide association mapping and haplotype analysis using CIMMYT Asia association mapping (CAAM) panel. The genomic regions associated with charcoal rot resistance identified were validated using QTL mapping in two mapping populations, apart from identifying population-specific QTLs. Validated regions/markers will be further studied in breeding populations for possible deployment in the breeding pipelines.

## Materials and Methods

### Plant Material

A set of 396 lines from the CAAM panel that were developed and adapted in Asian environments, involving inbred lines with tolerance to abiotic stresses like drought, high temperature, and excess moisture, besides quality protein maize (QPM) lines, and inbred lines derived from downy mildew-resistant populations in Asia, was used in genome-wide association study (GWAS). The CAAM panel included lines that are adapted to tropical, subtropical, lowland, mid-altitude, and highland environments and was classified into early maturing, intermediate maturing, and late maturing based on growing degree days (GDD). Most of the lines had yellow/orange kernel color, with very few lines had white kernel color (Supplementary Table 1).

Two biparental F_2:3_ families were formed to perform linkage mapping analysis for the validation of GWAS results. The first population (MSR) derived from a cross between a charcoal rot-resistant female parent CML495 and a susceptible male parent CML474 comprised 190 F_2:3_ families. CML495 is an elite lowland adapted, late inbred line with white kernel color. The second population (FMSR) derived from a cross between a resistant female parent WLS-F36-4-2-2-B-1-B^*^9 (now released as CML578) and a susceptible male parent CML474 comprised 257 F_3_ families. The common susceptible parent CML474 is an Asia-lowland adapted early line used as the early generation tester for heterotic group A.

### Phenotypic Evaluation

#### Screening Sites

The CAAM panel was evaluated under artificial inoculation conditions for charcoal rot at two hot spot locations: Borlaug Institute for South Asia (BISA) farm, Ludhiana, Punjab, India (30°55′ N, 75°54′ E; 229 masl; 750–800 mm/year rainfall) during the wet season of 2013 and International Crop Research Institute for Semi-Arid Tropics (ICRISAT) farm, Hyderabad, Telangana, India (17.53° N; 78.27° E.; 545 masl; 784 mm/year average rainfall) during the dry season of 2013 and 2014. For linkage mapping, F_2:3_ families of two mapping populations, MSR and FMSR, were evaluated for charcoal rot at ICRISAT farm, Hyderabad, during the dry season of 2017 and 2018, respectively. All disease evaluation trials were planted in alpha lattice design with two replications of a single row. The row length was 2 m with a spacing of 0.20 m between plant to plant and 0.75 m between row to row. Standard agricultural practices were maintained throughout the cropping season.

#### Inoculum Preparation and Inoculation Technique

Toothpick method was followed for artificial inoculation of the trials (Lal and Singh, 1984). In this method, mass multiplication of *M. phaseolina* for artificial inoculation was done on wooden toothpicks by the method proposed by Jardine and Leslie (1992), with slight modifications. For inoculum multiplication, wooden toothpicks were saturated in tap water for 12–15 h followed by air drying. Dried toothpicks (~250) were packed in 250 ml glass bottles with 50 ml distilled water and were autoclaved at 15 lbs and 121°C for 15 min. After sterilization, excess water was poured out of the glass bottles and potato dextrose broth (PDB) was added, followed by autoclaving at the same temperature and pressure regime. After cooling, freshly subcultured fungi were inoculated into the bottles under aseptic conditions and incubated at 25°C till the toothpicks were covered up with fungal growth (~15 days).

At the tassel emergence stage of the plants, colonized toothpicks were inserted into the stalks. This was attained by drilling a hole of 4–5 cm at 45° angle in the second internode (first elongated node) with an iron needle having a wooden handle, where the toothpicks were introduced into the hole.

#### Disease Scoring

Disease scores were taken after 45–50 days of inoculation by splitting the stalk of the inoculated plants. Longitudinally divided stalks were individually scored on disease severity on a 1–9 scale (Payak and Sharma, 1983), where a score of 1 = 25% infection of the inoculated node; 2 = 26–50% of infection in the inoculated node; 3 = 51–75% of infection in the inoculated node; 4 = 76–100% of infection in the inoculated node; 5 = lesser than 50% of infection in the adjacent node, 6 = more than 50% of infection in the adjacent node; 7 = infection in more than three nodes; 8 = infection in more than four nodes; and 9 = infection in five nodes or plant lodging due to disease. Disease scores 1–2 were rated as highly resistant (HR), 2.1–4 were rated as resistant (R), 4.1–6 were rated as moderately resistant (MR), and >6.1 were rated as susceptible (S). In each row, at least 10 plants were inoculated, and each inoculated plant was scored to obtain a mean disease score for the plot.

#### Phenotypic Data Analysis

Descriptive statistics like mean, skewness, kurtosis, and genetic correlation were estimated using Meta-R (Alvarado et al., 2015). The CR disease data were skewed toward susceptibility in the CAAM panel. Best linear unbiased prediction (BLUPs) was obtained using the software Meta−6.0 across year data analysis, and the single year data were used for GWAS and QTL mapping analysis, respectively. The linear models are implemented in *lmer* from package *lme4* of R (R Core Team 2013) using REML to calculate BLUPs and estimate variance components. Broad-sense heritability of the combined analysis across years was estimated as *H*^2^ = $\sigma_{\text{g}}^{2}$ /($\sigma_{\text{g}}^{2}$ + $\sigma_{\text{ge}}^{2}$/*e* + $\sigma_{\text{e}}^{2}$/*er*), where $\sigma_{\text{g}}^{2}$, $\sigma_{\text{ge}}^{2}$, and $\sigma_{\text{e}}^{2}$ are the genotypic, genotype-by-year interaction, and error variance components, respectively, and *e* and *r* are the number of years and number of replicates within each year included in the corresponding analysis, respectively.

#### DNA Isolation and Genotyping of CAAM Panel

Genomic DNA of the maize lines in the association mapping panel was isolated from leaves of 3–4-week-old seedlings (CIMMYT., 2001). Genotyping of the panel was performed at the Institute for Genomic Diversity, Cornell University, Ithaca, NY, USA, for single-nucleotide polymorphism (SNP) using genotyping by sequencing method (GBS). Genomic DNA was digested with the restriction enzyme Ap*eKI*. The GBS libraries were constructed in 96-plex and sequenced in Ilumina HiSeq 2000 (Elshire et al., 2011), and SNP calling was performed using TASSEL GBS pipeline (Glaubitz et al., 2014), where the GBS 2.7 sequences were used to anchor reads to the Maize B73 RefGen_v2 reference genome (www.maizegdb.org). Imputation was performed using FILLIN method in TASSEL 5.0, using GBS 2.7 haplotype files from Panzea (www.panzea.org) made from 8,000-site windows, as described in Swarts et al. (2014). The partially imputed GBS SNP data that had 955,690 genotypic data points (SNPs) across all the chromosomes were based on an algorithm that explores the closest neighbor in a small SNP window across the whole genome, permitting 5% mismatch (Romay et al., 2013). GWAS was conducted using 296,497 SNPs that were generated with the filtration criteria of call rate ≥ 0.7 and minor allele frequency (MAF) ≥ 0.05.

#### GWAS and Haplotype Regression

Methods studied for GWAS analysis were naïve model, where genotypic data were used without correction (*G*-test); general linear model (GLM), where genotypic data were corrected for structure (Q) using 10 principal components (G + Q-test); and single locus mixed linear model (MLM), where genotypic data were corrected for both structure and kinship (K) (G + Q + K) to avoid spurious associations. Additive models were used for *G*-test and GLM, and mixed model single locus (EMMAX) (Kang et al., 2010) was used for MLM for association studies in SVS version 8.6.0 (Golden Helix, Inc., Bozeman, MT, www. goldenhelix.com). The mixed association mapping model used was *Y* = SNP^*^β + PC^*^α + *K* ^*^μ + ε, where *Y* = response of the dependent variable (MSR Score), SNP = SNP marker (fixed effects), PC = principal component coordinate from the PCA (fixed effects), *K* = kinship matrix (random effects), α = vector of PC, β and μ = vectors of SNP and K, respectively, and ε = the error. A kinship matrix was estimated from identity-by-state distances matrix as executed in SVS version 8.6.0, where IBS distance = (no. of markers IBS2) + 0.5 × (no. of markers IBS1) no. of non-missing markers, where IBS1 and IBS2 are the states in which the two inbred lines share one or two alleles at a marker (Bishop and Williamson, 1990). Linkage disequilibrium (LD) was estimated on adjacent pairwise *r*^2^-values between adjacent SNPs among the SNPs from the GBS data and physical distances between those SNPs as described in Rashid et al. (2020). Manhattan and quantile–quantile plots were created using the association results. *P-*value threshold was estimated by using genome-wide LD between SNPs and the effective number of independent markers. Markers that were in approximate linkage equilibrium with each other were determined based on SNP pruning with LD *r*^2^ threshold of 0.1 to select a subset of markers representing linkage blocks, and the suggestive*-*value threshold to control the genome-wide error rate was 5.16 × 10^−5^ (Mao et al., 2015; Cui et al., 2016). SNPs with *P* ≤ 0.01 in GWAS of CAAM panel were selected for haplotype detection and trait regression. Expectation maximization (EM) algorithm (Excoffier and Slatkin, 1995) with 50 EM iterations, EM convergence tolerance of 0.0001, and a frequency threshold of 0.01 were used to estimate haplotype frequency as applied in SVS version 8.6.0. Block defining algorithm (Gabriel et al., 2002) was used to identify haplotype blocks to minimize historical recombination. Regression analysis was carried out with the haplotype blocks identified on the MSR BLUP values based on stepwise regression with forward elimination.

#### Linkage Map Construction and Quantitative Trait Loci Mapping

Genomic DNA of the F_2:3_ lines of mapping population was extracted from the 3–4 weeks old seedlings. Markers were selected across the genome from the Illumina Goldengate assay for the QTL mapping study, apart from a few GWAS-identified SNPs. The lines were genotyped with KASP assays developed from random and GWAS-identified SNP markers at LGC Genomics, London. Based on parental line polymorphism, MSR mapping population was genotyped with a set of 125 markers, and the second population, FMSR, was genotyped with a set of 166 SNPs. Linkage map was constructed using QTL IciMapping version 3.4 software using the twin criterion of more than 3.0 LOD and a maximum distance of 40 cm between two loci. The QTLs were identified for BLUPs of the disease score using inclusive composite interval mapping (ICIM) as implemented in QTL IciMapping version 3.4. The walking step in QTL scanning was 1 cm, and a likelihood odds (LOD) threshold of 3.138 and 3.460 was used to declare QTL in MSR and FMSR populations, respectively, which was based upon 1,000 times permutations analysis. QTL statistics were also reported for those in which the LOD score exceeded 2.5. The sign of the additive effect of each QTL was used to identify the origin of the favorable allele.

## Results

### Phenotypic Evaluation for Charcoal Rot Resistance

The CAAM panel consisting of 396 inbred lines was screened for charcoal rot resistance across three locations/years in India. The panel showed elevated disease severity, with a maximum score of 9.00 on a scale of 1.00–9.00 during all 3 years at two locations. Minimum disease scores of 2.10, 2.00, and 3.77 were observed at BISA, Ludhiana and Hyderabad, during years 1 and 2, respectively. The average disease score across locations was 7.21, which was skewed toward susceptibility. Broad-sense heritability (*h*^2^) was moderate to high (0.54–0.67) across single location with significant genotypic variance (*P* ≤ 0.001). QTL mapping population, MSR, evaluated at Hyderabad showed a trial mean of 5.62, with minimum and maximum disease scores of 3.76 and 7.79, respectively. The second mapping population, FMSR, showed an average trial mean of 6.21, with minimum and maximum scores of 3.42 and 8.90, respectively. Heritability estimates of MSR and FMSR trials were high, with 0.65 in MSR and 0.71 in FMSR population (Table 1). The response of both mapping populations showed continuous distribution for CR disease severity ranging from disease resistant or tolerant to susceptible reaction (Supplementary Figure 1). BLUPs were estimated to further conduct GWAS for charcoal rot resistance in association mapping panel and linkage mapping analysis.

**Table 1:** Summary statistics of CIMMYT Asia association mapping panel evaluated at three environments and two F_2:3_ linkage mapping populations evaluated during the dry season of 2017 and 2018.

**Location/year**	**Mean**	**Min**	**Max**	**Phenotypic variance**	**Error variance**	**Genotypic variance**	**G × E variance**	**Heritability**
ICRISAT-13	7.71	2.00	9.00	2.57	1.67	1.73^**^	**–**	0.67
ICRISAT-14	7.17	3.77	9.00	1.46	1.35	0.79^**^	**–**	0.54
BISA-13	6.71	2.10	9.00	1.23	1.01	0.73^**^	**–**	0.59
Across	7.25	4.51	9.00	0.73	1.89	0.41^**^	0.00016^**^	0.57
MSR-MP	5.62	3.76	7.79	0.59	0.42	0.38^**^	**–**	0.65
FMSR-MP	6.21	3.42	8.90	0.84	0.49	0.60^**^	**–**	0.71

** *P < 0.001*.

### GWAS for Resistance to Charcoal Rot

**Figure 1:**
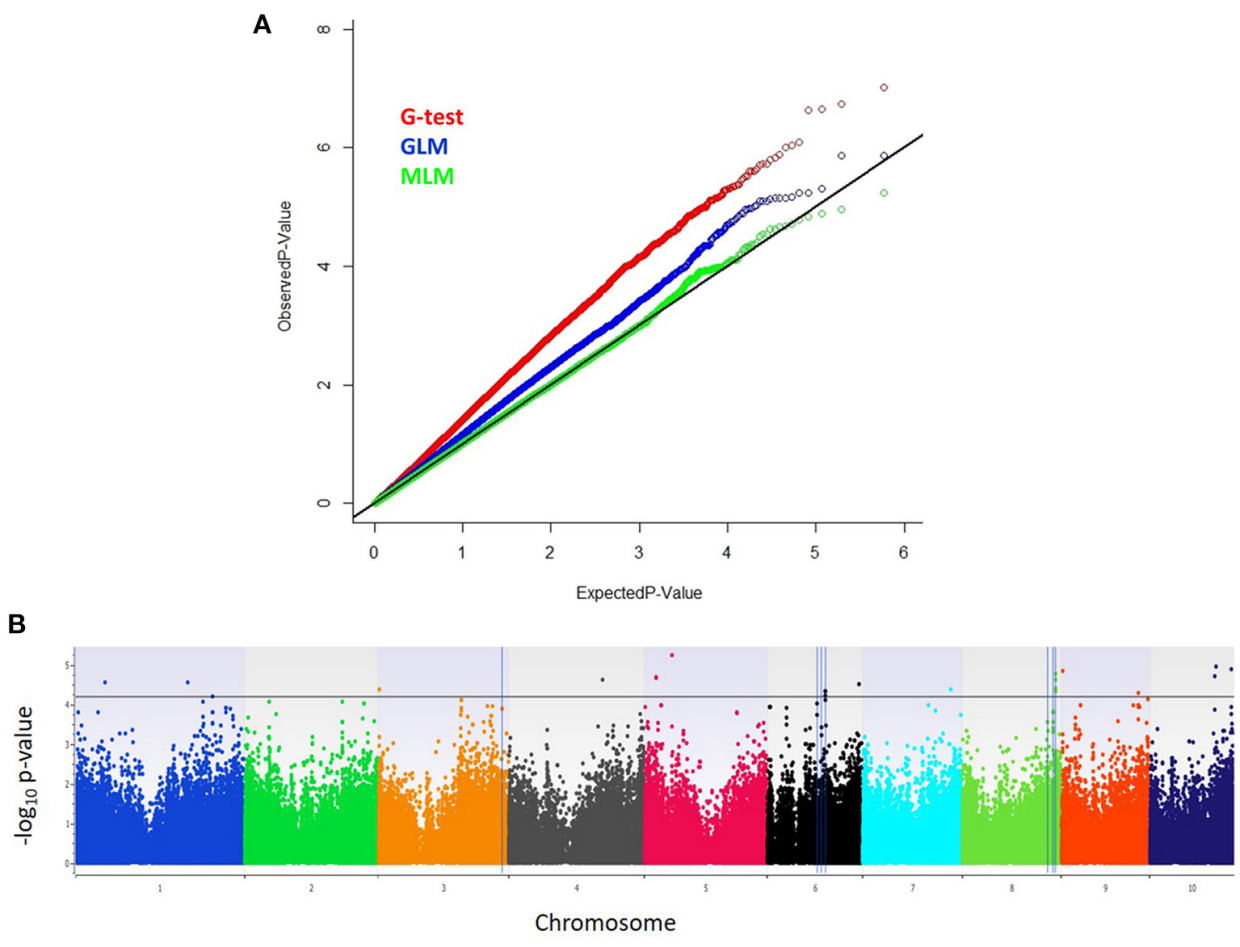
**(A)** Inflation depicted by Q–Q plots of observed vs. expected –log_10_ (*P*-values) plots for charcoal rot using the naïve association model (*G*-test), GLM (G + Q), and MLM (G + Q + K); G = genotype (fixed), Q = 10 principal components (fixed), K = kinship matrix (random) for CAAM panel. **(B)** Highly significant SNPs identified from MLM model using Manhattan plot, plotted with the individual SNPs on the *X*-axis and –log_10_ *P*-value of each SNP on the *Y*-axis. The horizontal line showed the cutoff *P*-value, and the vertical line represents the identified QTLs and haplotype blocks in these regions for charcoal resistance.

From high density imputed 955K GBS genotypic data, a subset of 296,497 SNPs fulfilling the criteria of call rate ≥0.7 and MAF ≥ 0.05 was used for conducting GWAS analysis. The quantile–quantile (QQ) plot with observed against expected –log_10_ *P*-value revealed that highest genomic inflation was observed in Naïve or *G*-test association model, followed by general linear model (GLM) or G+Q model, where genomic inflation was controlled with population structure using first 10 principal components (PCs). However, mixed linear model (MLM) or G + Q+K model corrected for both population structure (Q) and kinship (K) sighted minimum genomic inflation as noticed in the QQ plots (Figure 1). Therefore, highly significant associations for charcoal rot resistance in the CAAM panel were determined based on MLM analysis. The narrow-sense heritability for charcoal rot resistance due to the associated SNPs was found to be 0.53. The total number of SNPs identified to be linked with charcoal rot resistance was 19 with *P*-value ranging from 5.88 × 10^−06^ to 4.80 × 10^−05^ (Figure 1). The most significant association detected for resistance to charcoal rot was with SNP S5_48504604 on chromosome 5, which showed the lowest *P*-value, followed by SNP S10_117560618 on chromosome 10. Among the 19 SNPs detected, groups of SNPs located at close physical co-ordinates were found on chromosome 5 (S5_19528704, S5_19528705), chromosome 6 (S6_103513337 and S6_103513378), and chromosome 8 (S8_165726551, S8_165726553, S8_165726556, and S8_165726574) (Table 2). Based on the physical position of the significant SNPs with respect to B73 version 2 of the reference genome (http://ensembl.gramene.org/Zea_mays), the significant SNPs identified in GWAS were associated with 12 genes, several of which had functional domains involved in resistance to biotic stresses.

**Table 2:** Significantly associated single-nucleotide polymorphisms (SNPs) along with the predicted gene model and their function detected by genome-wide association studies in CIMMYT association mapping panel for charcoal rot resistance.

**Marker**	**Ch**	***P*-Value**	**PVE*%***	**Favorable allele**	**Predicted gene model**	**Gene name/best matching ortholog**	**Plants**	**Reported function**	**References**
S5_48504604	5	5.88 × 10^−06^	5.63	G	–	–	–	–	
S10_117560618	10	1.12 × 10^−05^	5.3	A	GRMZM2G072513	OSJNBa0088K19.7-like protein	Rice	–	
S10_144684808	10	1.29 × 10^−05^	5.22	G	GRMZM2G136895	*Zea mays* Beta-D-xylosidase 4	*Arabidopsis*, other plants	Cell wall modification, fruit development	Itai et al., 2003; Minic et al., 2004; Liao et al., 2012
S9_1994787	9	1.44 × 10^−05^	5.17	G	GRMZM2G500051	–	–	–	
S8_165726556	8	1.68 × 10^−05^	5.09	C	GRMZM2G414696	–	–	–	
S10_115937334	10	1.97 × 10^−05^	5.01	A	GRMZM2G050647	Exocyst complex component SEC5	*Arabidopsis*, other plants	Plant-pathogen interaction	Du et al., 2018
S5_19528704	5	2.11 × 10^−05^	4.97	G	GRMZM2G178767	*Zea mays* Dof zinc-finger protein DOF5.7	Plants	Abiotic stress, biotic stress	Sakamoto et al., 2004; Guo et al., 2009
S5_19528705	5	2.19 × 10^−05^	4.95	A					
S4_167190764	4	2.37 × 10^−05^	4.92	A	GRMZM2G168337	*Zea mays* Nicastrin	*Arabidopsis*, maize	Promotes maturation and proper trafficking of complex components and substrate recognition, biotic stress	Wang et al., 2012; Smolarkiewicz et al., 2014
S8_165726551	8	2.38 × 10^−05^	4.91	C	GRMZM2G414696	–	–	–	–
S1_52605386	1	2.85 × 10^−05^	4.82	T	–	–	–	–	–
S1_200489143	1	2.90 × 10^−05^	4.81	T	GRMZM2G557453	–	–	–	–
S6_163106367	6	3.07 × 10^−05^	4.78	A	AC206312.3_FGT008	–	–	–	–
S8_165726574	8	3.98 × 10^−05^	4.65	A	GRMZM2G414696	–	–	–	–
S3_2125663	3	4.31 × 10^−05^	4.61	T	GRMZM2G170047	*Zea mays* Cytochrome P450 71A26	Maize, wheat, barley	Oxidation- reduction reaction, defense mechanism, secondary metabolite synthesis, Fusarium head blight	Morant et al., 2003; Irmisch et al., 2015; Gunupuru et al., 2018
S7_156114994	7	4.34 × 10^−05^	4.61	G	GRMZM2G465999	*Zea mays* G-type lectin S-receptor-like serine/threonine-protein kinase B120	Plants	Biotic and abiotic stress tolerance, plant defense	Lannoo and Van Damme, 2014
S6_103513378	6	4.62 × 10^−05^	4.57	C	GRMZM2G122172	Aldehyde dehydrogenase family 2 member C4	Plants	Abiotic and biotic stresses tolerance	Wen et al., 2012; Brocker et al., 2013
S6_103513337	6	4.73 × 10^−05^	4.56	A					
S8_165726553	8	4.80 × 10^−05^	4.55	A	GRMZM2G414696	–	–	–	

*Ch, chromosome, PVE, Phenotypic varinace explained*.

### Haplotype Detection and Regression Analysis

Two hundred and eighty-nine SNPs (with *P* ≤ 10^−3^) that were identified in GWAS analysis were used to construct 44 haplotype blocks across 10 chromosomes, which were used in haplotype regression (HTR) analysis on estimated BLUP values. HTR analysis identified 21 haplotypes with Bonferroni *P* ≤ 0.05, which explained 3.22–6.48% of phenotypic variance. Haplotype blocks for charcoal rot resistance were identified on chromosomes 1, 2, 3, 5, 6, 8, and 9, formed with 2–8 SNPs (Table 3). Hap_8.1 on chromosome 8 formed by two SNPs, S8_151908973 and S8_151908983, showed the highest significance (Bonferroni *P-*value 7.73 × 10^−05^), followed by the Hap_5.2 on chromosome 5 (*P-*value 5.11 × 10^−06^ and Bonferroni *P*-value 2.24 × 10^−04^) (Table 3).

**Table 3:** Significant haplotypes identified in the CAAM panel for resistance to charcoal rot using haplotype regression.

**Haplotype block**	**Ch**	**Markers**	***P*-Value**	**PVE%**	**Bonferroni *P*-value**	**FDR**	**Favorable alleles**
Hap_1.1	1	S1_228148457, S1_228148501, S1_228148728, S1_228148775	0.000394055	4.4725128	0.017338434	0.001238	TCGG
Hap_1.2		S1_245219474, S1_245219477, S1_245221215, S1_245221351, S1_245221353, S1_245221363	0.000434489	4.14338	0.019117509	0.001275	CCCAAG
Hap_1.3		S1_259778254, S1_259778269	3.49E-05	4.6908192	0.001537657	0.000384	CG
Hap_1.4		S1_268948971, S1_268948972, S1_268948974, S1_268948981	0.000289605	4.3917062	0.012742613	0.001274	ATCC
Hap_1.5		S1_290819006, S1_290819008	8.31E-06	5.8851488	0.000365777	0.000122	GA
Hap_2.1	2	S2_212365693, S2_212365694	0.000543506	3.8516348	0.023914282	0.001407	AG
Hap_3.1	3	S3_148298879, S3_148298896, S3_148298906, S3_148299049	0.000180157	4.3758201	0.007926917	0.000991	GCCG
Hap_3.2		S3_168367332, S3_168367335, S3_168367337	0.00053713	3.7499107	0.02363372	0.001477	CGG
Hap_3.3		S3_202114642, S3_202114644	0.000666384	3.2229271	0.029320889	0.001466	GC
Hap_3.4		S3_220734668, S3_220734677	0.000547048	3.3326993	0.024070091	0.001337	CA
Hap_5.1	5	S5_19528704, S5_19528705, S5_19590454	4.59E-05	6.3666231	0.002019445	0.000404	CCA
Hap_5.2		S5_68423958, S5_68423980	5.11E-06	5.8413714	0.000224844	0.000112	CG
Hap_5.3		S5_194559998, S5_194560001, S5_194560045, S5_194560047, S5_194560048	0.001015383	3.2534765	0.044676862	0.002127	AATTA
Hap_6.1	6	S6_95934506, S6_95934536	0.000332882	3.5967653	0.014646826	0.001127	GT
Hap_6.2		S6_103513337, S6_103513340, S6_103513378, S6_103513510	0.00017213	4.3611641	0.00757371	0.001082	CGGG
Hap_8.1	8	S8_151908973, S8_151908983	1.76E-06	6.4890618	7.73E-05	7.73E-05	GT
Hap_8.2		S8_161523161, S8_161523199, S8_161523202, S8_161523204, S8_161523205, S8_161523207, S8_161523208, S8_161523210	0.000271353	4.1801244	0.011939511	0.001327	GACTCTCT
Hap_9.1	9	S9_24597525, S9_24597528, S9_24597531, S9_24597534	4.96E-05	4.5259142	0.002180251	0.000363	CTTG
Hap_9.2		S9_34173064, S9_34173069, S9_34173103	0.000311075	4.3479104	0.013687318	0.001141	GGC
Hap_9.3		S9_137258399, S9_137258400, S9_137258402	0.00057319	3.4234421	0.025220351	0.001327	GTG
Hap_9.4		S9_137258446, S9_137258482	0.000291508	4.0121756	0.012826349	0.001166	TA

*Ch, chromosome, PVE, Phenotypic varinace explained, FDR, False discovery rate*.

### Linkage Mapping for Charcoal Resistance

**Figure 2:**
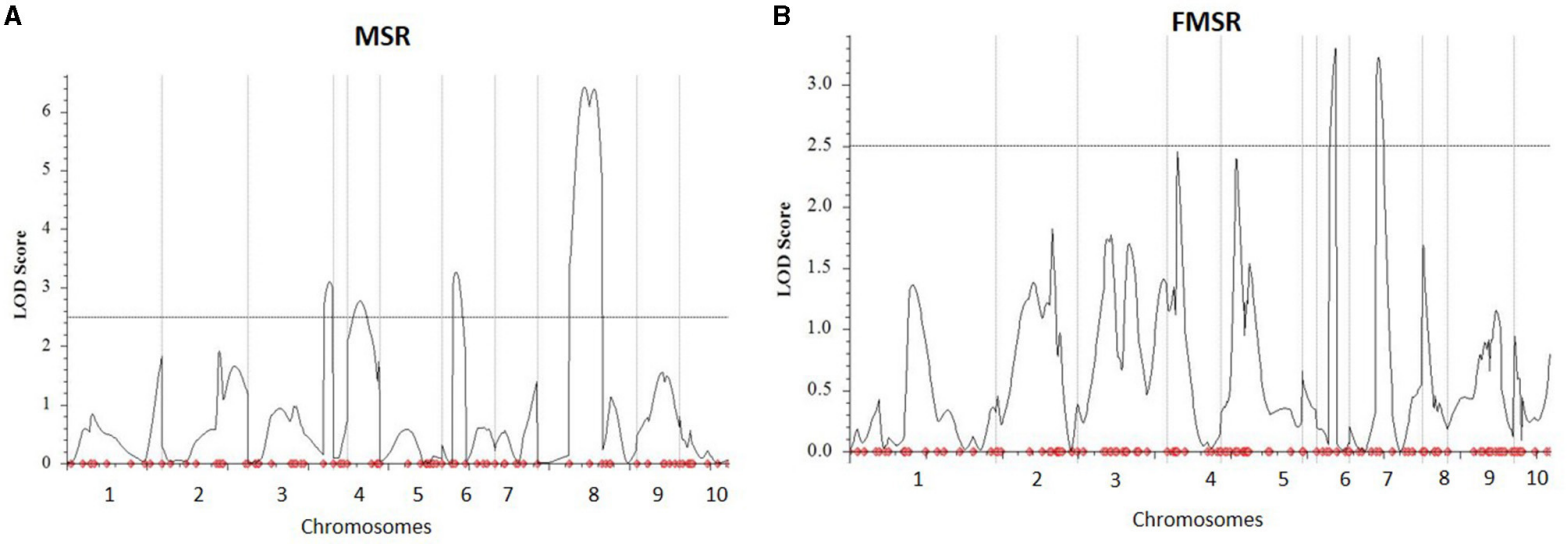
Plot of LOD scores from quantitative trait loci (QTL) analysis for charcoal rot resistance across 10 maize chromosomes in two F2: 3 biparental populations, **(A)** MSR and **(B)** FMSR. MSR and FMSR populations were evaluated under artificial inoculation conditions by *Macrophomina phaseolina*. The horizontal line represents the threshold LOD value of 2.5.

Two biparental mapping populations phenotyped for charcoal rot at Hyderabad, India, were used for QTL mapping and validation of the genomic regions identified through GWAS and HTR analysis. By genotyping the MSR and FMSR populations with 125 and 166 markers, respectively, linkage maps were constructed. The average marker densities for MSR and FMSR populations were 7.09 and 5.59 cm, respectively, across the 10 chromosomes. Inclusive composite interval mapping in MSR mapping population identified two QTLs on chromosomes 6 and 8 (Figure 2), and two other QTLs were detected on chromosomes 3 and 4 at the lower default LOD threshold of 2.5. QTL qMSR8 on chromosome bin 8.06–07 between markers PZA01964_29 and PHM4757_14 had the largest effect, which explained 13.86% of the phenotypic variation. Resistant alleles were contributed by the resistant parent CML495 for all the QTLs identified in MSR population. In the FMSR mapping population, no QTLs were detected at the LOD threshold of 3.460, and two QTLs were identified on chromosomes 6 and 7 (Figure 2) at a lower default threshold of 2.5. QTL qFMSR6 on chromosome bin 6.03–04 between the markers PZA01029_1 and S6_103513510 showed the largest effect explaining 6.56% of the phenotypic variance (Table 4). For the two QTLs, resistant alleles were contributed by the resistant parent (WLS-F36-4-2-2-B-1-B^*^9). QTLs qMSR6 and qFMSR6, identified on chromosome 6, were found to be overlapping based on the physical coordinates, and this region was identified in both GWAS and HTR analysis also. QTLs detected in the two mapping populations predominantly showed dominant effects; however, two QTLs detected in MSR mapping population showed additive effects for charcoal rot resistance.

**Table 4:** Quantitative trait loci (QTL) detected on different chromosomes by inclusive composite interval mapping analysis for resistance to charcoal rot in two F_2:3_ biparental mapping populations.

**Population**	**QTL**	**Ch**	**Ch bin**	**Position, cm**	**Marker interval**	**Physical position (B73_V2) (LM)**	**Physical position (RM)**	**LOD**	**PVE (%)**	**Total PVE%**	**Additive effect**	**Dominant effect**	**D/A**	**Gene action**
MSR	qMSR3	3	3.09	101	PZA03391_1–ZA00316_10	219,859,920	223,513,639	3.1	5.72	23.54	−0.1724	0.007	−0.04	A
	qMSR4	4	4.02–4.03	15	PHM3963_33–PHM259_7	5,459,125	14,326,036	2.77	6.49		−0.143	−0.1522	1.06	D
	**qMSR6**	**6**	**6.02–6.03**	**17**	**PHM12904_7–S6_103513378**	**88,691,499**	**103,513,378**	**3.26**	**5.65**		**−0.1685**	**0.0012**	**−0.01**	**A**
	**qMSR8**	**8**	**8.06–8.07**	**59**	**PZA01964_29–PHM4757_14**	**166,984,405**	**151,452,567**	**6.42**	**13.86**		**−0.2651**	**−0.0721**	**0.27**	**PD**
FMSR	qFMSR6	6	6.03–6.04	26	PZA01029_1–S6_103513510	114,031,392	103,513,510	3.298	6.56	10.48	−0.2115	−0.0393	0.185	D
	qFMSR7	7	7.03	37	PZA02643_1–PZA03166_1	128,365,318	137,632,654	3.223	6.51		−0.2068	0.0357	−0.17	D

*Ch, chromosome; LOD, likelihood odds; PVE, phenotypic variation explained; A, additive effect; D, dominant effect; PD, partially dominant*.

*The QTLs represented in bold show QTLs that had LOD scores above the LOD threshold based on 1,000 permutations, and the ones in normal fonts are the ones that were above LOD threshold of 2.5*.

## Discussion

Post-flowering stalk rots are complex diseases, due to collective infection with multiple soil-borne pathogens, intensified by abiotic stresses like drought and further compounded by secondary infections. Charcoal rot, caused by soil-borne pathogen *M. phaseolina*, is an important component of the PFSRs and its management methods include cultural practices, fungicide application, biological control, and resistant varieties. A comprehensive understanding of the host plant resistance is necessary to develop and deploy elite, stress-resistant varieties with little yield reduction in the presence of biotic stresses. As there are no reported studies on resistance to charcoal rot resistance in maize, we undertook this study to discover and validate genomic regions controlling this trait. A GWAS was conducted using a mapping panel that included tropical/subtropical inbred lines from CIMMYT breeding programs in Asia, Mexico, Kenya, Zimbabwe, and Colombia that are also acclimatized to the Asian tropics. The CAAM panel was previously used to study traits like resistance to sorghum downy mildew (Rashid et al., 2018), northern leaf corn blight (Rashid et al., 2020), and root traits under drought conditions (Zaidi et al., 2016) in Asian environments. Phenotypic evaluation of CAAM panel for charcoal rot at Hyderabad and Ludhiana, revealed that the panel was skewed toward susceptibility, possibly because both these locations had ideal environment for pathogen infection and spread, and the artificial inoculation using the toothpick method reduced the chances of escapes. The toothpick method has been widely used for artificial inoculation of stalk rots due to its simplicity and low cost (Tesso et al., 2009). In this study, we used linkage mapping apart from GWAS to study the genomic regions conferring resistance to charcoal rot. The high disease score mean in the AM panel compared with the mapping populations showed that the allele frequency of the resistant alleles might be lesser in the AM panel, whereas in the mapping populations such alleles contributed by the resistant parents were segregating in the populations, and hence higher allele frequency and lesser disease incidence.

Linkage mapping targets genetic recombination generated in artificially controlled crosses and offers huge advantages in terms of QTL detection power. However, it has the disadvantages of low mapping resolution, allele sampling, and speed. Unlike linkage mapping, GWAS makes use of the ancestral recombination events in a natural population to analyze marker-phenotype relations (Rafalski, 2002). It has the advantage of increased mapping resolution and speed but could have a lesser power of mapping (Korte and Farlow, 2013). Whereas, QTL mapping in biparental populations segregating for the relevant alleles at the associated/linked locus may be used in the validation of trait association (Rafalski, 2010), it also identifies novel QTLs not identified in GWAS, if the alleles are rare in the AM panel and/or the allelic phase differs across population structure groups (Famoso et al., 2011). To complement the GWAS analysis carried out in the Asia-adapted AM panel, two mapping populations, MSR and FMSR, with a common susceptible parent were used for linkage mapping to identify novel QTLs and for the validation of detected marker associations. A common susceptible early maturing parent, CML474, was used in both the mapping populations because it is highly susceptible to this disease and is being used as a tester for early maturity heterotic pool A. Markers spread across the genome were used for the QTL mapping study, along with some GWAS-identified SNP-based markers. There was no prior information on the status of the QTLs present in either of the parents. We conducted inclusive composite interval mapping to detect trait QTLs that were contributed by either of the parents. The QTLs identified that were co-located with the SNPs identified in GWAS were considered as validated in independent studies. The GWAS-SNPs that were not co-located within QTL intervals were not considered as unvalidated, as they might just not be segregating in the parental combinations studied.

Two QTLs were detected with PVE ranging from 5.65 to 13.86% in one of the populations. Apart from these, three QTLs were detected at a lower threshold from the two populations. The results indicated that phenotypic variation for charcoal rot resistance in the two populations was explained largely by minor to moderate effect alleles. This is in accordance with QTL studies of a number of complex traits in maize. Out of the two QTLs identified in MSR populations, qMSR6 was found to overlap, based on physical co-ordinates, with qFMSR6, a minor QTL identified at a lower threshold of 2.5 in the FMSR population. This assumes immense significance as it is not very common to observe stable QTLs for complex traits contributed by unrelated parental lines in different experiments. Among the genomic regions identified in GWAS, two SNPs on chromosome 6.03 (S6_103513337, S6_103513378) co-located with these QTLs were detected in linkage mapping. The trait-associated SNPs were located in the gene GRMZM2G122172, having the functional domain of aldehyde dehydrogenase (ALDH) family 2 member C4 (Carbon 4). Studies showed that ALDH upregulation is a common target of stress response pathway activation in plants, where ALDH responds to abiotic stresses leading to altered expression under exposure to stresses like drought (Bartels and Sunkar, 2005, Kotchoni et al., 2006). This function implies direct significance under charcoal rot infection, as the disease severity is directly related to drought stress. Also, studies have shown that ALDH gene from Chinese wild grapevine enhanced resistance to mildew pathogens and salt stress in *Arabidopsis* (Wen et al., 2012). Thus, this region can be considered as a region of interest for charcoal resistance and calls for further studies on dissection of the QTL and possible use in breeding populations. Another haplotype block, Hap_6.1 located at 95.93 Mb, was also identified within the QTL interval of qMSR6 identified in MSR mapping population.

On chromosome bin 8.06, four SNPs, S8_165726556, S8_165726551, S8_165726574, and S8_165726553, were identified, which co-located with the largest QTL identified in this study, qMSR8, located in the physical interval of 151.45 to 166.98 Mb on chromosome 8. Haplotype regression analysis identified two significant haplotypes (Hap_8.1 and Hap_8.2) for this trait within this QTL interval. In published studies on resistance to GSR, a major QTL *Rgsr*8.1 was also fine mapped to 2.04 Mb interval between 164.69 and 166.72 Mb, with two candidate resistant genes, one of which was an auxin-responsive element and the other encoding a disease resistance protein (Chen et al., 2017). Also, co-incident with Hap_8.1, Ma et al. (2017) identified a QTL for resistance to GSR at physical position between 146.4 and 158.9 Mb. It is interesting to note that our study identified and validated a genomic region contributing for resistance to charcoal rot, which also houses QTLs for resistance to another stalk rot pathogen, *Fusarium graminearum*, causing GSR. Apart from this, chromosomal bin 8.05–8.06 is known to harbor genes for resistance to multiple biotic stresses and is considered as one of the “complex, important and interesting” genomic regions in terms of maize disease resistance (Chung et al., 2010). Similar to this region on chromosome 8, trait-associated SNPs were identified on other chromosomes too that were located within previously mapped QTL intervals for GSR. Hap_3.4 was located within the QTL interval of minor QTL qMSR3 on chromosomal bin 3.09 at 220.73 Mb, where Ma et al. (2017) identified a QTL between 217.9 and 225.6 Mb for GSR resistance. In the same study, a major QTL *q*R*fg*3, at a physical position of 176.8–209.9 Mb, was detected across three field trials on chromosome bin 3.6/07 explaining 10.7–19.4% phenotypic variance for GSR resistance. The haplotype regression analysis for charcoal rot in this study identified Hap_ 3.3 on chromosome bin 3.07 at a physical position of 202.11 Mb, which fell within the QTL interval of QTL *q*R*fg*3. Ma et al. (2017) also identified a QTL on chromosome 5 between 49.9 and 152.0 Mb for GSR resistance, which also housed Hap_5.2 located at a physical co-ordinate of 68.42 Mb in this study. Further studies on gene characterization at these loci for both these diseases will be required to understand if common resistance mechanisms operate toward resistance to multiple stalk rot pathogens.

Several significant trait-associated SNPs identified in the GWAS were located within genes with functional domains related to biotic and abiotic stress tolerance, immune response, metabolism, plant development, and maturity (Table 2). Two SNPs, S5_19528704 and S5_19528705 identified for charcoal resistance and located in the same chromosomal bin as a QTL identified for GSR resistance on chromosome 5.02-5.04 (Pè et al., 1993) were located within the predicted gene GRMZM2G178767 that codes for a *Zea mays* Dof zinc-finger protein DOF5.7, which is implicated in abiotic and biotic stress tolerance in plants (Guo et al., 2009, Sakamoto et al., 2004). Zinc-finger domain is present in a well-known class of plant-resistant proteins, NBS-LRR, that are involved in effector-triggered immune response (Gupta et al., 2012). Zinc-finger-based WRKY transcription factor (TF) plays a broad and pivotal role in plant immune responses (Eulgem et al., 2007). Another significant SNP, S3_2125663, was located in the gene GRMZM2G170047 that potentially codes for cytochrome P450, which is known to boost disease resistance. Cytochrome P450s are membrane-bound enzymes that can accomplish oxidation–reduction reactions (Morant et al., 2003) and are involved in plant defense and secondary metabolite synthesis in classical xenobiotic detoxification pathway (Schuler and Werck-Reichhart, 2003). It was also reported to play a major role in resistance to Fusarium head blight disease caused by *Fusarium graminearum* in wheat (Walter et al., 2008; Walter and Doohan, 2011). Similarly, the gene GRMZM2G168337, which houses SNP S4_167190764, was implicated in the synthesis of Nicastrin, which was found to be upregulated in maize after inoculation with southern corn rust (Wang et al., 2012). A gene where a charcoal rot-associated SNP S7_156114994 was located is GRMZM2G465999, which is a type of lectin S-receptor-like serine/threonine-protein kinase. Plant kinases constitute a diverse protein superfamily, which is capable of recognizing and interacting with specific carbohydrate structures either from invading microorganisms or deformed plant cell wall structures, and plant lectin motifs are used constantly to combat against pathogens and predators during plant defense (Lannoo and Van Damme, 2014). Gene GRMZM2G050647 associated with SNP S10_115937334 codes for exocyst complex component SEC5, which plays a role in plant–pathogen interaction. Exocyst complex is a conserved multiprotein complex that has eight subunits that are used in pathogen defense against hemi-biotrophic pathogens like *Phytophthora infestans* and *Pseudomonas syringae*, and some exocyst subunits can act as a susceptibility factor for necrotrophic pathogens like *Botrytis cinerea*. (Du et al., 2018). In *Arabidopsis*, Exo70B mutants showed lesion-mimic cell death mediated by salicylic acid accumulation (Kulich et al., 2013).

## Conclusion

The genetic architecture of charcoal rot resistance was dissected through association and linkage mapping. Nineteen SNPs were found to be highly significant for charcoal rot resistance in GWAS analysis, and haplotype regression identified 21 haplotypes, of which Hap_8.1 at 151.90 Mb on chromosome 8 was shown to have the most significant effect on the trait. Inclusive composite interval mapping in two F_2:3_ mapping populations detected QTLs on chromosomes 6 and 8 with PVE ranging from 5.65 to 13.86%. QTLs on chromosome bin 6.03, with a flanking marker at 103.51 Mb, were detected in both the linkage mapping populations, albeit at a lower threshold in one of the populations. SNPs/haplotypes in this QTL interval were identified in the GWAS and haplotype regression studies also. Similarly, the SNPs and haplotype detected on chromosome 8 were also validated in QTL mapping in one mapping population. These haplotypes on chromosomes 6 and 8 can be further analyzed in breeding populations for the possible deployment of trait markers for charcoal rot resistance. Several significant SNPs and haplotypes identified in this study were found to be located within published QTL intervals for GSR resistance. To our understanding, this study is the first report for mapping and validating genomic regions for charcoal rot resistance in maize.

## Data Availability Statement

The datasets presented in this study can be found in online repositories. The names of the repository/repositories and accession number(s) can be found at: https://data.cimmyt.org/dataset.xhtml?persistentId=hdl:11529/10548606.

## Author Contributions

SN designed the experiment. ZR, HK, VB, PS, and SH generated the phenotyping data. ZR and VB analyzed the data. SN and ZR wrote the manuscript. All authors reviewed the manuscript.

## Conflict of Interest

The authors declare that the research was conducted in the absence of any commercial or financial relationships that could be construed as a potential conflict of interest.

## Publisher's Note

All claims expressed in this article are solely those of the authors and do not necessarily represent those of their affiliated organizations, or those of the publisher, the editors and the reviewers. Any product that may be evaluated in this article, or claim that may be made by its manufacturer, is not guaranteed or endorsed by the publisher.
