# Supplementary material for: Identification and Validation of Genomic Regions Associated With Charcoal Rot Resistance in Tropical Maize by Genome-Wide Association and Linkage Mapping
Source: Front Plant Sci. 2021 Oct 8;12:726767. doi: 10.3389/fpls.2021.726767 (PMC8531636; doi:10.3389/fpls.2021.726767)

**Identification and validation of genomic regions associated with charcoal rot resistance in tropical Maize by genome-wide association and linkage mapping.**

Zerka Rashid^1^, Harleen Kaur^2^, Veerendra Babu^1^, Pradeep Kumar Singh^1^, Sharanappa I Harlapur^3^ and Sudha Krishnan Nair^1^*

**^1^**International Maize and Wheat Improvement Center (CIMMYT), ICRISAT Campus, Patancheru, Greater Hyderabad 502324, Telangana, India

^2^Punjab Agriculture University, Ludhiana, 141004, Punjab, India

^3^ University of Agricultural Sciences. Krishi Nagar Dharwad, 580005, Karnataka, India

*Corresponding author: [Sudha.nair@cgiar.org](mailto:Sudha.nair@cgiar.org)

Supplementary table 1: List of CIMMYT Asia Association Mapping panel lines with growing degree days (GDD), Kernel colour, texture and origin of germplasm.

| **Sr No.** | **Sample ID** | **Region of Origin** | **Kernel colour** | **Texture** | **GDD** |
| --- | --- | --- | --- | --- | --- |
| 1 | CIMIND2_1 | Latin America | Orange | Flint | 889 |
| 2 | CIMIND2_2 | Latin America | Orange | Flint | 889 |
| 3 | CIMIND2_26 | Latin America | Orange | Flint | 845 |
| 4 | CIMIND2_74 | Mexico | Orange | Flint | 815 |
| 5 | CIMIND2_86 | Mexico | Yellow | Flint | 830 |
| 6 | CIMIND2_51 | Mexico | Yellow | Flint | 889 |
| 7 | CIMIND2_6 | Mexico | Orange | Flint | 1023 |
| 8 | CIMIND2_66 | Mexico | Yellow | Flint | 1037 |
| 9 | CIMIND2_90 | Mexico | Yellow | Flint | 964 |
| 10 | CIMIND2_7 | Zimbabwe | White | Flint | 933 |
| 11 | CIMIND2_55 | _ | Yellow | Flint | 964 |
| 12 | CIMIND2_91 | Mexico | Yellow | Flint | 876 |
| 13 | CIMIND2_8 | Mexico | Yellow | Flint | 876 |
| 14 | CIMIND2_9 | Mexico | Orange | Flint | 1023 |
| 15 | CIMIND-101 | Mexico | White | Flint | 917 |
| 16 | CIMIND-121 | Mexico | Orange | Flint | 1063 |
| 17 | CIMIND-123 | Mexico | Yellow | Flint | 1051 |
| 18 | CIMIND-128 | Mexico | Orange | Flint | 1051 |
| 19 | CIMIND-135 | Mexico | Orange | Flint | 1051 |
| 20 | CIMIND-148 | Mexico | White | Flint | 1010 |
| 21 | CIMIND-149 | Mexico | Orange | Flint | 876 |
| 22 | CIMIND-160 | Mexico | White | Flint | 1023 |
| 23 | CIMIND-166 | Mexico | White | Flint | 1037 |
| 24 | CIMIND-171 | Mexico | White | Flint | 1063 |
| 25 | CIMIND-188 | Mexico | Orange | Flint | 830 |
| 26 | CIMIND-205 | Zimbabwe | White | Flint | 902 |
| 27 | CIMIND-206 | Zimbabwe | White | Flint | 1076 |
| 28 | CIMIND-218 | Mexico | Orange | Flint | 789 |
| 29 | CIMIND-222 | Mexico | Orange | Flint | 861 |
| 30 | CIMIND-226 | Mexico | Orange | Flint | 1037 |
| 31 | CIMIND-23 | Mexico | White | Flint | 917 |
| 32 | CIMIND-232 | Zimbabwe | White | Flint | 1037 |
| 33 | CIMIND-233 | Mexico | White | Flint | 1051 |
| 34 | CIMIND-235 | Mexico | White | Flint | 1104 |
| 35 | CIMIND-239 | Mexico | White | Flint | 1051 |
| 36 | CIMIND-242 | Mexico | White | Flint | 1051 |
| 37 | CIMIND-243 | Mexico | White | Flint | 1051 |
| 38 | CIMIND-244 | Mexico | White | Flint | 1037 |
| 39 | CIMIND-245 | Zimbabwe | White | Flint | 1037 |
| 40 | CIMIND-246 | Zimbabwe | White | Flint | 902 |
| 41 | CIMIND-249 | Asia | White | Flint | 917 |
| 42 | CIMIND-250 | Mexico | White | Flint | 1037 |
| 43 | CIMIND-252 | Zimbabwe | White | Flint | 1037 |
| 44 | CIMIND-253 | Zimbabwe | White | Flint | 933 |
| 45 | CIMIND-254 | Zimbabwe | White | Flint | 917 |
| 46 | CIMIND-255 | _ | Orange | Flint | 1076 |
| 47 | CIMIND-257 | Mexico | Yellow | Flint | 830 |
| 48 | CIMIND-258 | Asia | Orange | Flint | 889 |
| 49 | CIMIND-261 | Asia | Orange | Flint | 830 |
| 50 | CIMIND-262 | Asia | Orange | Flint | 876 |
| 51 | CIMIND-263 | Asia | Orange | Flint | 801 |
| 52 | CIMIND-264 | Asia | Yellow | Flint | 876 |
| 53 | CIMIND-265 | Asia | Orange | Flint | 889 |
| 54 | CIMIND-266 | Asia | Orange | Flint | 889 |
| 55 | CIMIND-267 | Mexico | Orange | Flint | 876 |
| 56 | CIMIND-268 | Mexico | Orange | Flint | 845 |
| 57 | CIMIND-269 | Mexico | Orange | Flint | 917 |
| 58 | CIMIND-271 | Mexico | Orange | Flint | 979 |
| 59 | CIMIND-272 | Zimbabwe x Mexico | Orange | Flint | 1051 |
| 60 | CIMIND-273 | Zimbabwe x Mexico | Orange | Flint | 917 |
| 61 | CIMIND-274 | Mexico | Orange | Flint | 889 |
| 62 | CIMIND-275 | Asia | Orange | Flint | 845 |
| 63 | CIMIND-279 | Asia | Orange | Flint | 889 |
| 64 | CIMIND-280 | Asia | Orange | Flint | 801 |
| 65 | CIMIND-283 | Mexico | Orange | Flint | 876 |
| 66 | CIMIND-284 | Mexico | Orange | Flint | 964 |
| 67 | CIMIND-285 | Mexico | Orange | Flint | 1051 |
| 68 | CIMIND-286 | Mexico | Orange | Flint | 1051 |
| 69 | CIMIND-288 | Mexico | Yellow | Flint | 1023 |
| 70 | CIMIND-289 | Mexico | Yellow | Dent | 1051 |
| 71 | CIMIND-290 | Mexico | Yellow | Flint | 1063 |
| 72 | CIMIND-291 | Mexico | Orange | Flint | 933 |
| 73 | CIMIND-292 | Mexico | Orange | Flint | 1037 |
| 74 | CIMIND-293 | Mexico | Orange | Flint | 938 |
| 75 | CIMIND-294 | Mexico | Orange | Flint | 1023 |
| 76 | CIMIND-295 | Mexico | Orange | Flint | 1117 |
| 77 | CIMIND-296 | Mexico | Yellow | Flint | 1104 |
| 78 | CIMIND-297 | Mexico | Yellow | Flint | 1051 |
| 79 | CIMIND-298 | Mexico | Orange | Flint | 1063 |
| 80 | CIMIND-299 | Mexico | Yellow | Flint | 1037 |
| 81 | CIMIND-300 | Mexico | Yellow | Flint | 1023 |
| 82 | CIMIND-301 | Asia | Orange | Flint | 876 |
| 83 | CIMIND-302 | Asia | Orange | Flint | 789 |
| 84 | CIMIND-303 | Asia | Orange | Flint | 815 |
| 85 | CIMIND-304 | Asia | Orange | Flint | 830 |
| 86 | CIMIND-305 | Mexico | Orange | Flint | 1051 |
| 87 | CIMIND-306 | Mexico | Orange | Flint | 845 |
| 88 | CIMIND-307 | Mexico | Orange | Flint | 979 |
| 89 | CIMIND-309 | Asia | Orange | Flint | 902 |
| 90 | CIMIND-310 | Asia | Orange | Flint | 845 |
| 91 | CIMIND-311 | Asia | Orange | Flint | 876 |
| 92 | CIMIND-312 | Asia | Orange | Flint | 861 |
| 93 | CIMIND-314 | _ | Orange | Flint | 789 |
| 94 | CIMIND-315 | _ | Orange | Flint | 933 |
| 95 | CIMIND-316 | _ | Orange | Flint | 876 |
| 96 | CIMIND-317 | _ | Orange | Flint | 789 |
| 97 | CIMIND-318 | _ | Orange | Flint | 845 |
| 98 | CIMIND-319 | _ | Orange | Flint | 830 |
| 99 | CIMIND-320 | Mexico | Orange | Flint | 889 |
| 100 | CIMIND-321 | Zimbabwe | White | Flint | 876 |
| 101 | CIMIND-322 | Zimbabwe | White | Flint | 902 |
| 102 | CIMIND-323 | Zimbabwe | White | Flint | 845 |
| 103 | CIMIND-324 | Zimbabwe | White | Flint | 876 |
| 104 | CIMIND-325 | Zimbabwe | White | Flint | 861 |
| 105 | CIMIND-326 | Zimbabwe | White | Flint | 845 |
| 106 | CIMIND-327 | Mexico | White | Flint | 861 |
| 107 | CIMIND-328 | Mexico | White | Flint | 1051 |
| 108 | CIMIND-329 | Mexico | White | Flint | 1051 |
| 109 | CIMIND-330 | Asia | Orange | Flint | 876 |
| 110 | CIMIND-331 | Asia | Yellow | Flint | 861 |
| 111 | CIMIND-332 | Mexico | Orange | Flint | 1023 |
| 112 | CIMIND-333 | Mexico | Orange | Flint | 1037 |
| 113 | CIMIND-334 | Mexico | Orange | Flint | 876 |
| 114 | CIMIND-335 | Mexico | Orange | Flint | 889 |
| 115 | CIMIND-337 | Asia | Orange | Flint | 1037 |
| 116 | CIMIND-338 | Asia | Orange | Flint | 1051 |
| 117 | CIMIND-339 | Asia | Orange | Flint | 861 |
| 118 | CIMIND-340 | Asia | Orange | Flint | 917 |
| 119 | CIMIND-341 | Asia | Orange | Flint | 917 |
| 120 | CIMIND-342 | _ | Yellow | Flint | 830 |
| 121 | CIMIND-345 | Mexico | Orange | Flint | 889 |
| 122 | CIMIND-346 | Mexico | Orange | Flint | 861 |
| 123 | CIMIND-347 | Asia | Orange | Flint | 1023 |
| 124 | CIMIND-349 | Mexico | Yellow | Flint | 889 |
| 125 | CIMIND-358 | Mexico | White | Flint | 1037 |
| 126 | CIMIND-361 | Mexico | Orange | Flint | 1037 |
| 127 | CIMIND-363 | Mexico | Orange | Flint | 1023 |
| 128 | CIMIND-364 | Mexico | Orange | Flint | 830 |
| 129 | CIMIND-366 | Asia | Yellow | Flint | 902 |
| 130 | CIMIND-367 | Asia | Orange | Flint | 917 |
| 131 | CIMIND-368 | Asia | Orange | Flint | 1037 |
| 132 | CIMIND-370 | Asia | Orange | Flint | 876 |
| 133 | CIMIND-371 | Asia | Orange | Flint | 951 |
| 134 | CIMIND-372 | Asia | Orange | Flint | 789 |
| 135 | CIMIND-374 | Asia | Orange | Flint | 876 |
| 136 | CIMIND-378 | Mexico | Orange | Flint | 1010 |
| 137 | CIMIND-380 | Mexico | Orange | Flint | 861 |
| 138 | CIMIND-383 | Mexico | Orange | Flint | 889 |
| 139 | CIMIND-384 | Mexico | Orange | Flint | 917 |
| 140 | CIMIND-385 | Mexico | Orange | Flint | 1051 |
| 141 | CIMIND-386 | Mexico | Yellow | Flint | 1063 |
| 142 | CIMIND-388 | Mexico | Yellow | Flint | 1051 |
| 143 | CIMIND-390 | Mexico | Orange | Flint | 1037 |
| 144 | CIMIND-391 | Mexico | Yellow | Flint | 1023 |
| 145 | CIMIND-395 | Mexico | Yellow | Flint | 917 |
| 146 | CIMIND-399 | Mexico | Orange | Flint | 889 |
| 147 | CIMIND-40 | Mexico | White | Flint | 1037 |
| 148 | CIMIND-401 | Mexico | Orange | Flint | 1037 |
| 149 | CIMIND-402 | Mexico | Orange | Flint | 1037 |
| 150 | CIMIND-405 | Mexico | Orange | Flint | 889 |
| 151 | CIMIND-406 | Mexico | Yellow | Flint | 845 |
| 152 | CIMIND-408 | Mexico | Yellow | Flint | 902 |
| 153 | CIMIND-41 | Mexico | Orange | Flint | 1023 |
| 154 | CIMIND-411 | Mexico | Orange | Flint | 1037 |
| 155 | CIMIND-412 | Mexico | Orange | Flint | 902 |
| 156 | CIMIND-416 | Mexico | Orange | Flint | 1051 |
| 157 | CIMIND-420 | Mexico | Orange | Flint | 1010 |
| 158 | CIMIND-422 | Mexico | Orange | Flint | 995 |
| 159 | CIMIND-423 | Mexico | Orange | Flint | 876 |
| 160 | CIMIND-424 | Mexico | Orange | Flint | 1037 |
| 161 | CIMIND-425 | Mexico | Orange | Flint | 917 |
| 162 | CIMIND-426 | Mexico | Orange | Flint | 902 |
| 163 | CIMIND-427 | Mexico | Orange | Flint | 902 |
| 164 | CIMIND-428 | Mexico | Orange | Flint | 902 |
| 165 | CIMIND-429 | Mexico | Orange | Flint | 1037 |
| 166 | CIMIND-430 | Mexico | Yellow | Flint | 1063 |
| 167 | CIMIND-436 | Mexico | Orange | Flint | 889 |
| 168 | CIMIND-57 | Mexico | White | Flint | 1023 |
| 169 | CIMIND-62 | Mexico | White | Flint | 1037 |
| 170 | CIMIND-73 | Mexico | Orange | Flint | 1010 |
| 171 | CIMIND-74 | Mexico | Orange | Flint | 1010 |
| 172 | CIMIND-75 | Mexico | Orange | Flint | 1037 |
| 173 | CIMIND-76 | Mexico | Orange | Flint | 889 |
| 174 | CIMIND-77 | Mexico | Orange | Flint | 1037 |
| 175 | CIMIND-81 | Mexico | Yellow | Flint | 1037 |
| 176 | CIMIND-87 | Mexico | Yellow | Flint | 1037 |
| 177 | CIMIND-90 | Mexico | Orange | Flint | 1076 |
| 178 | CIMIND-91 | Mexico | Orange | Flint | 917 |
| 179 | CIMIND-93 | Mexico | Orange | Flint | 1037 |
| 180 | CIMIND-P8 | Asia | white | Flint | 1037 |
| 181 | CIMIND-P9 | Asia | Orange | Flint | 995 |
| 182 | CIMIND-V1 | Mexico | Orange | Flint | 845 |
| 183 | CIMIND-V10 | Mexico | Yellow | Flint | 917 |
| 184 | CIMIND-V101 | Mexico | Yellow | Flint | 889 |
| 185 | CIMIND-V102 | Mexico | Yellow | Flint | 889 |
| 186 | CIMIND-V104 | Mexico | Orange | Flint | 876 |
| 187 | CIMIND-V106 | Asia | Orange | Flint | 845 |
| 188 | CIMIND-V108 | Mexico | Yellow | Flint | 1104 |
| 189 | CIMIND-V109 | Asia | Orange | Flint | 801 |
| 190 | CIMIND-V11 | Mexico | Orange | Flint | 1063 |
| 191 | CIMIND-V110 | Asia | Orange | Flint | 830 |
| 192 | CIMIND-V111 | Asia | Orange | Flint | 845 |
| 193 | CIMIND-V112 | Mexico | Orange | Flint | 975 |
| 194 | CIMIND-V113 | Mexico | White | Flint | 1023 |
| 195 | CIMIND-V115 | Mexico | Orange | Flint | 902 |
| 196 | CIMIND-V116 | Asia | Orange | Flint | 830 |
| 197 | CIMIND-V118 | Mexico | Orange | Flint | 1023 |
| 198 | CIMIND-V119 | Asia | Orange | Flint | 845 |
| 199 | CIMIND-V12 | Mexico | Yellow | Flint | 1051 |
| 200 | CIMIND-V120 | Mexico | Orange | Flint | 902 |
| 201 | CIMIND-V121 | Asia | Orange | Flint | 876 |
| 202 | CIMIND-V122 | Asia | Orange | Flint | 902 |
| 203 | CIMIND-V123 | Asia | Orange | Flint | 876 |
| 204 | CIMIND-V124 | Asia | Orange | Flint | 861 |
| 205 | CIMIND-V125 | Mexico | White | Flint | 979 |
| 206 | CIMIND-V126 | Mexico | White | Flint | 1076 |
| 207 | CIMIND-V127 | Mexico | White | Flint | 1051 |
| 208 | CIMIND-V128 | Mexico | Orange | Flint | 1023 |
| 209 | CIMIND-V13 | Asia | Orange | Flint | 789 |
| 210 | CIMIND-V130 | Mexico | Orange | Flint | 861 |
| 211 | CIMIND-V131 | Asia | Orange | Flint | 876 |
| 212 | CIMIND-V132 | Mexico | Yellow | Flint | 1051 |
| 213 | CIMIND-V134 | Asia | Orange | Flint | 889 |
| 214 | CIMIND-V136 | Mexico | White | Flint | 902 |
| 215 | CIMIND-V137 | Mexico | White | Flint | 889 |
| 216 | CIMIND-V138 | Mexico | Orange | Flint | 870 |
| 217 | CIMIND-V139 | Asia | Orange | Flint | 902 |
| 218 | CIMIND-V140 | Asia | Orange | Flint | 889 |
| 219 | CIMIND-V141 | Asia | Orange | Flint | 876 |
| 220 | CIMIND-V142 | Mexico | Orange | Flint | 902 |
| 221 | CIMIND-V146 | Mexico | White | Flint | 815 |
| 222 | CIMIND-V147 | Mexico | Yellow | Flint | 876 |
| 223 | CIMIND-V148 | Mexico | Orange | Flint | 845 |
| 224 | CIMIND-V149 | Mexico | Orange | Flint | 845 |
| 225 | CIMIND-V15 | Mexico | Orange | Flint | 1037 |
| 226 | CIMIND-V150 | Mexico | Orange | Flint | 861 |
| 227 | CIMIND-V151 | Mexico | Yellow | Flint | 876 |
| 228 | CIMIND-V152 | Mexico | White | Flint | 1063 |
| 229 | CIMIND-V153 | Mexico | White | Flint | 830 |
| 230 | CIMIND-V154 | Mexico | Orange | Flint | 876 |
| 231 | CIMIND-V155 | _ | Yellow | Flint | 902 |
| 232 | CIMIND-V156 | Mexico | Yellow | Flint | 995 |
| 233 | CIMIND-V158 | Mexico | Yellow | Flint | 902 |
| 234 | CIMIND-V159 | Mexico | Orange | Flint | 948 |
| 235 | CIMIND-V160 | Mexico | Yellow | Flint | 917 |
| 236 | CIMIND-V161 | Mexico | White | Flint | 861 |
| 237 | CIMIND-V162 | Mexico | Orange | Flint | 845 |
| 238 | CIMIND-V163 | Mexico | Orange | Flint | 876 |
| 239 | CIMIND-V164 | Mexico | White | Flint | 889 |
| 240 | CIMIND-V165 | Kenya | White | Flint | 889 |
| 241 | CIMIND-V166 | Asia | Orange | Flint | 876 |
| 242 | CIMIND-V167 | Asia | Orange | Flint | 876 |
| 243 | CIMIND-V168 | Mexico | Orange | Flint | 889 |
| 244 | CIMIND-V169 | Mexico | Orange | Flint | 889 |
| 245 | CIMIND-V17 | Mexico | Orange | Flint | 1037 |
| 246 | CIMIND-V170 | Mexico | Orange | Flint | 995 |
| 247 | CIMIND-V171 | Mexico | Orange | Flint | 830 |
| 248 | CIMIND-V172 | Mexico | Orange | Flint | 889 |
| 249 | CIMIND-V173 | Mexico | Orange | Flint | 889 |
| 250 | CIMIND-V174 | Asia | Orange | Flint | 876 |
| 251 | CIMIND-V175 | Asia | Orange | Flint | 830 |
| 252 | CIMIND-V176 | Asia | Orange | Flint | 845 |
| 253 | CIMIND-V177 | Asia | Orange | Flint | 889 |
| 254 | CIMIND-V178 | Asia | Orange | Flint | 876 |
| 255 | CIMIND-V179 | Asia | Orange | Flint | 876 |
| 256 | CIMIND-V18 | Mexico | Yellow | Flint | 933 |
| 257 | CIMIND-V180 | _ | Yellow | Flint | 1037 |
| 258 | CIMIND-V181 | Asia | Orange | Flint | 815 |
| 259 | CIMIND-V183 | Asia | Orange | Flint | 889 |
| 260 | CIMIND-V184 | Asia | Orange | Flint | 1023 |
| 261 | CIMIND-V185 | Mexico | Orange | Flint | 902 |
| 262 | CIMIND-V186 | Mexico | Orange | Flint | 948 |
| 263 | CIMIND-V188 | Asia | Orange | Flint | 889 |
| 264 | CIMIND-V191 | Mexico | Yellow | Flint | 917 |
| 265 | CIMIND-V192 | Mexico | Orange | Flint | 861 |
| 266 | CIMIND-V194 | Zimbabwe | White | Flint | 995 |
| 267 | CIMIND-V195 | Zimbabwe | White | Flint | 902 |
| 268 | CIMIND-V196 | Mexico | Orange | Flint | 889 |
| 269 | CIMIND-V197 | Asia | Orange | Flint | 861 |
| 270 | CIMIND-V198 | Asia | Orange | Flint | 845 |
| 271 | CIMIND-V199 | Asia | Orange | Flint | 845 |
| 272 | CIMIND-V2 | Mexico | Orange | Flint | 801 |
| 273 | CIMIND-V20 | Mexico | Orange | Flint | 995 |
| 274 | CIMIND-V200 | Mexico | White | Flint | 1037 |
| 275 | CIMIND-V201 | Zimbabwe | White | Flint | 845 |
| 276 | CIMIND-V202 | Mexico | White | Flint | 1037 |
| 277 | CIMIND-V203 | Mexico | Orange | Flint | 815 |
| 278 | CIMIND-V204 | Mexico | White | Flint | 876 |
| 279 | CIMIND-V205 | Mexico | Orange | Flint | 815 |
| 280 | CIMIND-V206 | _ | Yellow | Flint | 830 |
| 281 | CIMIND-V208 | Mexico | Yellow | Flint | 902 |
| 282 | CIMIND-V209 | Mexico | Orange | Flint | 1010 |
| 283 | CIMIND-V21 | Mexico | Orange | Flint | 979 |
| 284 | CIMIND-V210 | Mexico | Orange | Flint | 889 |
| 285 | CIMIND-V211 | Mexico | Yellow | Flint | 876 |
| 286 | CIMIND-V212 | Mexico | Orange | Flint | 1023 |
| 287 | CIMIND-V215 | Mexico | Yellow | Flint | 1023 |
| 288 | CIMIND-V22 | Mexico | Orange | Flint | 917 |
| 289 | CIMIND-V222 | Mexico | Yellow | Flint | 902 |
| 290 | CIMIND-V224 | Mexico | Orange | Flint | 876 |
| 291 | CIMIND-V226 | Mexico | Orange | Flint | 861 |
| 292 | CIMIND-V231 | Asia | Orange | Flint | 830 |
| 293 | CIMIND-V232 | Asia | Orange | Flint | 861 |
| 294 | CIMIND-V234 | Mexico | Orange | Flint | 902 |
| 295 | CIMIND-V235 | Asia | Orange | Flint | 861 |
| 296 | CIMIND-V236 | Asia | Orange | Flint | 845 |
| 297 | CIMIND-V237 | Asia | Orange | Flint | 830 |
| 298 | CIMIND-V24 | Mexico | Yellow | Flint | 889 |
| 299 | CIMIND-V241 | Mexico | White | Flint | 876 |
| 300 | CIMIND-V242 | Mexico | White | Flint | 889 |
| 301 | CIMIND-V243 | Mexico | White | Flint | 1051 |
| 302 | CIMIND-V244 | Mexico | Yellow | Flint | 845 |
| 303 | CIMIND-V246 | Mexico | Orange | Flint | 845 |
| 304 | CIMIND-V248 | Mexico | Yellow | Flint | 995 |
| 305 | CIMIND-V25 | Mexico | Yellow | Flint | 933 |
| 306 | CIMIND-V252 | Mexico | Yellow | Flint | 845 |
| 307 | CIMIND-V253 | Mexico | Yellow | Flint | 902 |
| 308 | CIMIND-V254 | Mexico | Yellow | Flint | 938 |
| 309 | CIMIND-V255 | Mexico | Yellow | Flint | 845 |
| 310 | CIMIND-V257 | Mexico | Yellow | Flint | 861 |
| 311 | CIMIND-V259 | Mexico | Orange | Flint | 801 |
| 312 | CIMIND-V26 | Mexico | Orange | Flint | 902 |
| 313 | CIMIND-V260 | Mexico | Orange | Flint | 789 |
| 314 | CIMIND-V262 | Mexico | Orange | Flint | 830 |
| 315 | CIMIND-V265 | Mexico | White | Flint | 889 |
| 316 | CIMIND-V268 | Mexico | White | Flint | 801 |
| 317 | CIMIND-V27 | Mexico | Orange | Flint | 889 |
| 318 | CIMIND-V270 | Mexico | White | Flint | 789 |
| 319 | CIMIND-V272 | Mexico | White | Flint | 830 |
| 320 | CIMIND-V273 | Mexico | White | Flint | 845 |
| 321 | CIMIND-V275 | Mexico | Orange | Flint | 861 |
| 322 | CIMIND-V28 | Mexico | Orange | Flint | 861 |
| 323 | CIMIND-V29 | Mexico | Orange | Flint | 933 |
| 324 | CIMIND-V3 | Asia | Yellow | Flint | 789 |
| 325 | CIMIND-V30 | Mexico | Orange | Flint | 933 |
| 326 | CIMIND-V31 | Mexico | Orange | Flint | 1051 |
| 327 | CIMIND-V32 | Mexico | Orange | Flint | 889 |
| 328 | CIMIND-V33 | Mexico | Orange | Flint | 729 |
| 329 | CIMIND-V35 | Mexico | Orange | Flint | 1037 |
| 330 | CIMIND-V36 | Mexico | Orange | Flint | 1010 |
| 331 | CIMIND-V37 | Mexico | Orange | Flint | 1023 |
| 332 | CIMIND-V38 | Mexico | Orange | Flint | 815 |
| 333 | CIMIND-V39 | Mexico | Yellow | Flint | 889 |
| 334 | CIMIND-V4 | Asia | Orange | Flint | 845 |
| 335 | CIMIND-V40 | Mexico | Orange | Flint | 876 |
| 336 | CIMIND-V41 | Asia | Orange | Flint | 815 |
| 337 | CIMIND-V42 | Asia | Orange | Flint | 889 |
| 338 | CIMIND-V44 | Asia | Orange | Flint | 845 |
| 339 | CIMIND-V45 | Asia | Orange | Flint | 876 |
| 340 | CIMIND-V46 | Mexico | Orange | Flint | 830 |
| 341 | CIMIND-V47 | Mexico | Yellow | Flint | 845 |
| 342 | CIMIND-V49 | Brazil | Orange | Flint | 917 |
| 343 | CIMIND-V5 | Mexico | Orange | Flint | 789 |
| 344 | CIMIND-V50 | Asia | Orange | Flint | 889 |
| 345 | CIMIND-V51 | Asia | Orange | Flint | 889 |
| 346 | CIMIND-V52 | Mexico | Yellow | Flint | 861 |
| 347 | CIMIND-V53 | Asia | Orange | Flint | 845 |
| 348 | CIMIND-V54 | Asia | Orange | Flint | 876 |
| 349 | CIMIND-V55 | Asia | Orange | Flint | 876 |
| 350 | CIMIND-V56 | Asia | Orange | Flint | 951 |
| 351 | CIMIND-V57 | Asia | Orange | Flint | 902 |
| 352 | CIMIND-V58 | Mexico | Orange | Flint | 902 |
| 353 | CIMIND-V61 | Mexico | Orange | Flint | 1023 |
| 354 | CIMIND-V62 | Mexico | Orange | Flint | 889 |
| 355 | CIMIND-V65 | Mexico | Orange | Flint | 1076 |
| 356 | CIMIND-V66 | Mexico | Yellow | Flint | 1037 |
| 357 | CIMIND-V67 | Mexico | Orange | Flint | 1010 |
| 358 | CIMIND-V69 | Asia | Orange | Flint | 845 |
| 359 | CIMIND-V7 | Mexico | White | Flint | 1023 |
| 360 | CIMIND-V71 | Mexico | Orange | Flint | 964 |
| 361 | CIMIND-V72 | Asia | Orange | Flint | 1037 |
| 362 | CIMIND-V73 | Asia | Orange | Flint | 830 |
| 363 | CIMIND-V74 | Asia | Orange | Flint | 815 |
| 364 | CIMIND-V76 | Mexico | Yellow | Flint | 876 |
| 365 | CIMIND-V8 | Mexico | White | Flint | 995 |
| 366 | CIMIND-V80 | Asia | Orange | Flint | 815 |
| 367 | CIMIND-V81 | Asia | Orange | Flint | 845 |
| 368 | CIMIND-V83 | Mexico | White | Flint | 1037 |
| 369 | CIMIND-V84 | Zimbabwe | White | Flint | 1037 |
| 370 | CIMIND-V85 | Latin America | Yellow | Flint | 1037 |
| 371 | CIMIND-V86 | Mexico | Yellow | Flint | 889 |
| 372 | CIMIND-V87 | Mexico | Orange | Flint | 861 |
| 373 | CIMIND-V88 | Mexico | Orange | Flint | 861 |
| 374 | CIMIND-V89 | Mexico | Orange | Flint | 845 |
| 375 | CIMIND-V9 | Asia | Yellow | Flint | 876 |
| 376 | CIMIND-V91 | Mexico | White | Flint | 830 |
| 377 | CIMIND-V92 | Mexico | White | Flint | 801 |
| 378 | CIMIND-V94 | Mexico | White | Flint | 861 |
| 379 | CIMIND-V95 | Mexico | White | Flint | 861 |
| 380 | CIMIND-V96 | Asia | Orange | Flint | 1051 |
| 381 | CIMIND-V98 | Mexico | White | Flint | 889 |
| 382 | CIMIND-V99 | Mexico | Orange | Flint | 876 |
| 383 | DTMA-196 | Mexico | Orange | Flint | 1051 |
| 384 | DTMA-210 | Mexico | Orange | Flint | 876 |
| 385 | DTMA-234 | Mexico | Yellow | Flint | 876 |
| 386 | DTMA-240 | Mexico | Yellow | Flint | 995 |
| 387 | DTMA-276 | Mexico | Yellow | Flint | 845 |
| 388 | DTMA-280 | Mexico | Yellow | Flint | 845 |
| 389 | DTMA-281 | Mexico | Orange | Flint | 889 |
| 390 | DTMA-283 | Mexico | Yellow | Flint | 933 |
| 391 | DTMA-56 | Latin America | Orange | Flint | 861 |
| 392 | DTMA-65 | Latin America | Orange | Flint | 902 |
| 393 | DTMA-69 | Latin America | Orange | Flint | 876 |
| 394 | DTMA-70 | Latin America | Orange | Flint | 889 |
| 395 | DTMA-72 | Latin America | Orange | Flint | 889 |
| 396 | DTMA-78 | Latin America | Orange | Flint | 1063 |

Supplementary Fig.-1: Phenotypic distribution of charcoal rot severity values observed in two MSR and FMSR F_2:3_ mapping populations. Arrows indicate the performance of parents.


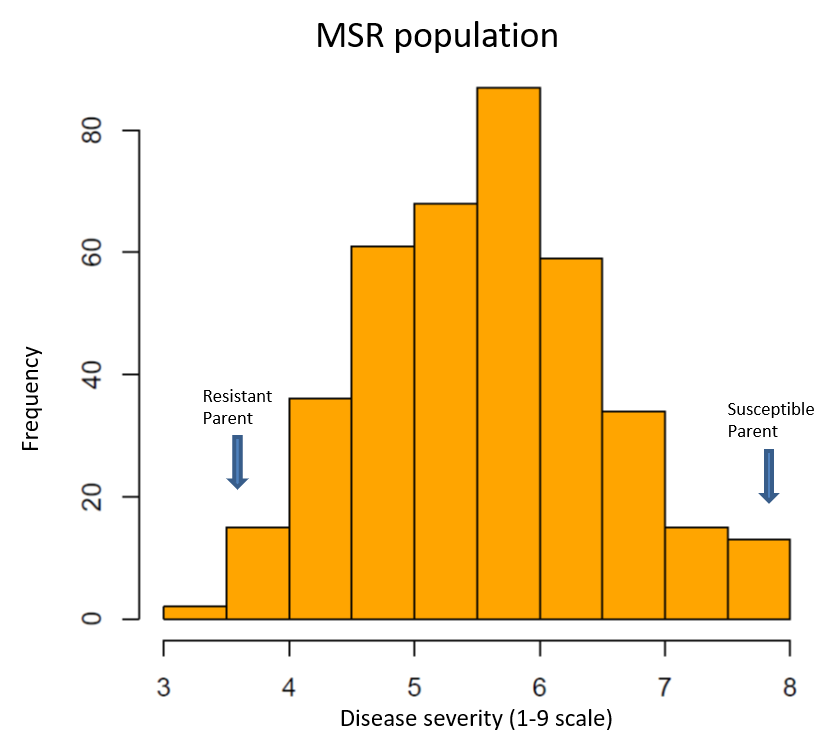


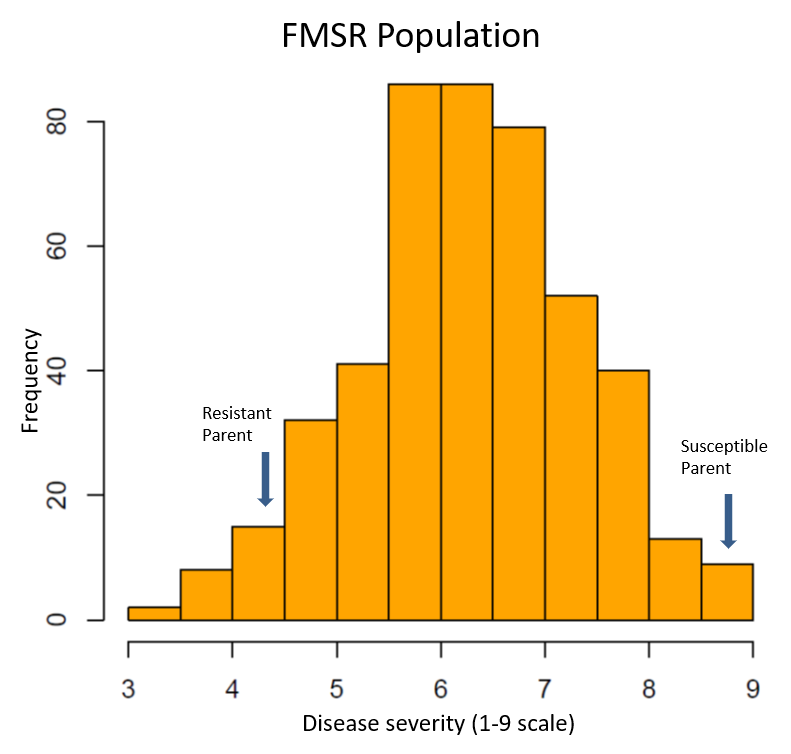

Supplement: Supplementary file 1 [file Data_Sheet_1.docx]
